# Assessment of the effectiveness of the CD3+ tool to detect counterfeit and substandard anti-malarials

**DOI:** 10.1186/s12936-016-1180-2

**Published:** 2016-02-25

**Authors:** JaCinta S. Batson, Daniel K. Bempong, Patrick H. Lukulay, Nicola Ranieri, R. Duane Satzger, Leigh Verbois

**Affiliations:** Food and Drug Administration, Forensic Chemistry Center, 6751 Steger Drive, Cincinnati, OH 45237 USA; United States Pharmacopeial Convention, 12601 Twinbrook Parkway, Rockville, MD 20852 USA; Food and Drug Administration, 10903 New Hampshire Avenue, Silver Spring, MD 20993 USA

**Keywords:** Anti-malarials, Counterfeit medicines, Medicine quality screening, Substandard medicines

## Abstract

**Background:**

The US FDA recently developed CD3+, a counterfeit detection tool that is based on sample illumination at specific wavelengths of light and visual comparison of suspect sample and packaging materials to an authentic sample. To test performance of the CD3+ in field conditions, a study was conducted in Ghana which compared the CD3+ side-by-side with two existing medicine quality screening technologies—TruScan™ Portable Raman spectrometer and GPHF Minilab^®^.

**Methods:**

A total of 84 anti-malarial test samples comprising artemether–lumefantrine tablets and artesunate–amodiaquine tablets were used. The technologies were evaluated for sensitivity in determining counterfeit/substandard (The term counterfeit or falsified is used in this article to refer to medicines that carry a false representation of identity or source or both. The term substandard is used to refer to medicines that do not meet the quality specifications given in the accepted pharmacopeia.) medicines, specificity in determining authentic products, and reliability of the results. Authentic samples obtained from manufacturers were used as reference standards. HPLC analysis data was used as the “gold standard” for decisions regarding a sample being authentic or substandard/counterfeit.

**Results:**

CD3+ had a sensitivity of 1.00 in detecting counterfeit/substandard products compared to Minilab (0.79) and TruScan (0.79). CD3+ had a lower specificity (0.53) in determining authentic products compared to the specificities reached by Minilab (0.99) and TruScan (1.00). High sensitivity in this context means that the technology is effective in identifying substandard/counterfeit products whereas the low specificity means that the technique can sometimes mischaracterize good products as substandard/counterfeit. Examination of dosage units only (and not packaging) using CD3+ yielded improved specificity 0.64. When only assessment of sample identification was done, the TruScan provided sensitivity (1.00) and specificity (0.99); and the Minilab provided sensitivity (1.00) and specificity (1.00). All three technologies demonstrated 100 % reliability when used to analyse the same set of samples over 3 days by a single analyst and also when used to determine the same set of samples by three different analysts. Eight of the field samples were confirmed to be counterfeits with no active pharmaceutical ingredient content. All three technologies identified these samples as counterfeits.

**Conclusions:**

The study revealed the relative effectiveness of the technologies as quality control tools. Using a combination of CD3+, with either the Minilab or TruScan, to screen for medicine quality will allow for complete examination of both the dosage units and the packaging to decide whether it is authentic or counterfeit.

## Background

The proliferation of counterfeit and substandard medicines has been a major concern to the global public health community. Medicine quality survey studies, particularly for anti-malarial medicines, have shown prevalence rate of counterfeit and substandard medicines ranging from 20 % to as high as 64 % [1–3]. Similar studies on quality of anti-tuberculosis and anti-retroviral medicines also revealed the extent of the problem of counterfeits and substandard products [4, 5]. Counterfeit and substandard anti-infective medicines pose a threat to public health and their prevalence has been linked to treatment failure, increased mortality and morbidity, and emergence of drug resistance [6, 7].

Preventing the circulation of counterfeit and substandard medicines has become a great challenge especially in countries with limited resources. In many developing countries, the national quality control laboratory of the medicines regulatory authority lacks the capacity to fully evaluate the quality of medicines on the market, usually due to the lack of equipment, personnel, and infrastructure needed for quality control testing. To help ameliorate the situation, particularly in resource-limited countries, a number of simple, non-sophisticated, and affordable medicine quality screening tools have been used or introduced for use in screening the quality of medicines. The use of the screening tools reduces the number of samples that a national medicines quality control laboratory must test, which subsequently reduces the burden on the national laboratory and its limited resources [8].

Two commonly used field-based screening tools are the Global Pharma Health Fund (GPHF) Minilab^1^ which is based on thin-layer chromatography and the TruScan^2^ hand-held Raman spectrometer from Thermo Scientific, which is based on Raman spectroscopy.

The United States Food and Drug Administration (US FDA) recently developed a counterfeit detection tool known as the CD3+. The tool is based on sample illumination at specific wavelengths of light and visual comparison of suspect packaging materials and dosage form to an authentic sample [9]. To test the performance of the CD3+ in field conditions, US FDA collaborated with the US Pharmacopeial Convention (USP) to conduct a field study in Ghana.

In the study, side-by-side comparison of CD3+ with two existing screening tools was performed on field samples (samples acquired in the markets of Ghana) to obtain an understanding of the sensitivity^3^, specificity^4^, and reliability^5^ associated with the use of these technologies. The study used commonly available anti-malarial medicines in Ghana to investigate the unique advantages of these screening tools.

The specific objectives of the study were as follows:

*Objective 1* Evaluate the sensitivity and specificity of the US FDA CD3+, the GPHF Minilab, and the Thermo Scientific TruScan hand-held Raman spectrometer in Ghana as counterfeit/substandard detection tools for two commonly used artemisinin-based combination therapy (ACT) medicines—artemether–lumefantrine (AR-LU) and artesunate–amodiaquine (AS-AQ)—for the treatment of malaria in sub-Saharan Africa.

*Objective 2* Evaluate the reliability of CD3+ to detect substandard and counterfeit medicines and compare it to the reliability of the GPHF Minilab and TruScan Raman hand-held spectrometer.

*Objective 3* Establish the comparative advantages and disadvantages of the three field based technologies.

## Methods

### Development of authentic libraries

Authentic samples of the anti-malarial products used in the study were sourced directly from their respective manufacturers (Table 1) and stored under conditions specified on the labelling. The CD3+ Authentic Image Library (CDAIL) was developed at the US FDA’s Forensic Chemistry Center (FCC). The CDAIL consisted of white light images of dosage forms and packaging, quadrant images of the packaging for printing process analysis, and images of the dosage forms and packaging at all the wavelengths and filter combinations featured on the tool. The Raman authentic spectral library for the TruScan was developed by the Promoting the Quality of Medicines (PQM) staff at USP headquarters using the authentic samples. In the case of GPHF Minilab analyses, the authentic samples were used as reference standards as Minilab does not have a library feature.

**Table 1:** Authentic samples used in the study

Product	Manufacturer	Strength (mg)	# of lots
Coartem (AR-LU) tablets	Novartis	20/120	5
Coartem dispersible tablets	Novartis	20/120	5
Artemether-Lumefantrine tablets	Ipca	20/120	4
Lumerax (AR-LU) tablets	Ipca	40/240	2
Winthrop Artesunate-Amodiaquine Amodiaquine tablets	Sanofi-Aventis	50/135	5
Winthrop Artesunate-Amodiaquine Amodiaquine tablets	Sanofi-Aventis	100/270	5
Artesunate-Amodiaquine tablets	Ipca	50/135	3
Artesunate-Amodiaquine tablets	Ipca	100/270	3
Total			32

*AR–LU* Artemether and Lumefantrine

### Field samples

Through the Promoting the Quality of Medicines (PQM) programme, funded by the United States Agency for International Development and implemented by USP, sentinel sites had been established in Ghana and five of these sentinel sites served as sample collection and field testing stations for this study. The field samples were collected by staff from the Ghana Food and Drugs Authority (Ghana FDA) at the sentinel sites located in the cities of Accra (ACC), Kumasi (KSI), Takoradi (TAK), Ho, and Bolgatanga (BOL), using convenience sampling—a non-probability sampling approach where samples that are the easiest to access are collected and collection areas are strategically selected. Field sample testing using the CD3+ and the GPHF Minilab were performed at these study sites. All other analyses, including HPLC analyses of field samples and authentic samples, TruScan Raman analyses, and other study testing activities, took place at USP’s Center for Pharmaceutical Advancement and Training (CePAT), a USP Global Health Impact Program located in Accra, Ghana. TruScan Raman analyses were not done in the field because there were insufficient units of the tool for all the study sites.

### HPLC analyses of authentic and field samples

HPLC analyses of authentic samples and field samples were performed and the results were used as the gold standard to judge all results obtained with the three screening tools. The HPLC procedures were sourced from the *USP Medicines Compendium* [10] and from a published article [11] and complete verification of the analytical methods were done before using the procedures. The HPLC analysis was based on analysis of the dosage unit only, since packaging and labelling could not be evaluated by this technique. The tests performed were identification and assay of the content of active pharmaceutical ingredients (API). The procedures used were not specific for determination of impurities associated with the APIs in the medicines; hence, impurity levels of the samples were not determined.

### Assessment of objective 1: sensitivity and specificity experiments

A total of 84 samples were used for the evaluation of the sensitivity and specificity of the three technologies (Table 2). This consisted of field samples, authentic samples, counterfeit samples, and authentic samples that had been stressed at 40 °C/75 % RH for 4 weeks in a stability chamber.

**Table 2:** Breakdown of test samples used to evaluate sensitivity and specificity of three technologies

Product	Manufacturer	Field samples				Additional samples				Total
		Accra	Bolga	HO	Kumasi	Takoradi	Counter–FEIT	Authentic	Stressed	
Artemetheter-Lumefantrine tablets, 20/120 mg	Ipca^a^	0	1	0	3	1	0	0	0	5
Lumerax (Artemether + Lumefantrine tablets, 40/240 mg)	Ipca	2	0	0	4	5	0	0	0	11
Coartem (Artemether + Lumefantrine tablets, 20/120 mg)	Novartis	2	4	3	3	2	3	0	0	17
Coartem dispersible (Artemether + Lumefantrine tablets, 20/120 mg)	Novartis	4	0	3	0	3	0	3	1	14
Winthrop (Artesunate + Amodiaquine tablets, 50/135 mg)	Sanofi-Aventis	8	5	1	1	10	0	3	1	29
Winthrop (Artesunate + Amodiaquine tablets, 100/270 mg)	Sanofi-Aventis	1	3	1	0	3	0	0	0	8
Total		17	13	8	11	24	3	6	2	84

^a^Ipca’s Artesunate-Amodiaquine tablets were not on the market at the time of the study

For CD3+ evaluation, the packaging material (primary and secondary packaging, and labelling), the printing on the packaging material, and the dosage units were visually examined at various wavelength/filter combinations to determine if the observed images were consistent with the library images of the authentic sample. Conclusions regarding whether or not a sample was consistent with the authentic were based on the results of all three examinations—packaging print, packaging material, and dosage unit.

In the case of GPHF Minilab testing, thin layer chromatography (TLC) analysis [12] of each sample was conducted using the corresponding authentic sample as the reference standard. The retardation factor (R_F_) values and intensities of the spots on the TLC plate—for the sample and the authentic product—were used to make decisions regarding identity and drug content of the sample. Pass or fail decisions were based on the results of both the identification and content analyses.

For the TruScan Raman analyses, the spectrometer was used to measure a Raman spectrum of the sample and the measured Raman spectrum was compared^6^ to that of the authentic Raman reference spectrum in the library. A pass decision was obtained based on statistical consistency (*p* > 0.05) of the sample spectrum with that of the authentic.

#### CD3+: use of library images

Study samples were compared with images in the CDAIL to determine if the sample images were consistent with (CW) or not consistent with (NCW) the library images, which is pass or fail.

#### CD3+: use of side-by-side image comparison

Samples were sent to the participants without their knowledge that the samples were authentic (“blind samples”), and the participants were asked to determine whether the samples were authentic or not. The blind sample study indicated that some authentic samples were flagged as not consistent with (NCW) the CDAIL. This prompted USP to setup another method of comparison by using side-by-side comparison of authentic and field sample. Thus, in addition to the library image comparison in the blind sample study, CePAT staff performed side-by-side comparisons of images of blind samples with that of a physical authentic sample to investigate if the use of library images alone contributed to the NCW results reported for authentic samples by the study sites.

#### Evaluation of the ability of the technologies to distinguish between different strengths of a product

Tests were performed to determine the ability of the screening technologies to distinguish between products presented in different strengths as reflected by API content. For this evaluation, the following authentic samples were used: AS-AQ (50/135 mg)(Sanofi, Maphar, Morocco), AS-AQ (100/270 mg)(Sanofi, Maphar, Morocco), AR-LU (20/120 mg)(Ipca Laboratories Ltd., India), AR-LU (40/240 mg) (Ipca Laboratories Ltd., India).

CD3+, GPHF Minilab, and TruScan analyses of the AS-AQ (50/135 mg) “sample” were performed using the higher strength product—AS-AQ (100/270 mg)—as the reference product. Results from the tools indicating whether or not the sample was consistent with the reference product were documented. These analyses were repeated, the second time using the higher strength product as “sample” and the lower strength product as “reference.” The two AR-LU samples were similarly analysed using the three technologies.

### Assessment of objective 2: reliability experiments

Reliability of results from the three technologies were assessed using samples consisting of three lots each of select AR-LU and AS-AQ authentic samples, samples stressed at 40 °C/75 % RH for 4 weeks, and counterfeit samples received from the Ghana FDA (Table 3). Three analysts each performed CD3+ analysis, GPHF Minilab analysis, and TruScan Raman analysis on all of these samples. One of the analysts repeated the test daily on three different days. The ability of each tool to give the same results with these repeated measurements was determined.

**Table 3:** List of samples used to evaluate reliability of three technologies

Sample type	Sample name	Strength (mg)	# of lots
Authentic	Coartem tablets	20/120	1
	Coartem Dispersible tablets	20/120	2
	Winthrop Artesunate-Amodiaquine tablets	50/135	2
	Winthrop Artesunate-Amodiaquine tablets	100/270	1
Counterfeit^a^	Coartem tablets	20/120	3
Stressed^b^	Coartem Dispersible tablets	20/120	1
	Winthrop Artesunate-Amodiaquine tablets	50/135	1
Total			11

^a^These samples were obtained from the Ghana FDA and had previously been tested and confirmed to be counterfeit

^b^Samples stressed at 40 °C/75 % RH for 4 weeks. HPLC analysis showed there were no significant changes in the content of the active ingredients of the stressed samples

## Results and discussions

### Objective 1 results: sensitivity and specificity

Sensitivity and specificity, in this study, are measures of how well a screening technology performs when it is compared to the “gold standard”—HPLC—in detecting counterfeit and authentic anti-malarial tablets. Sensitivity provides the probability that the technology will correctly detect a substandard/counterfeit product. Specificity, on the other hand, provides the probability that the technology will correctly identify a authentic product.

The sensitivity of a screening technology was calculated as the ratio of the total number of samples that are detected as substandard/counterfeit by the technology to the total number of samples that are substandard/counterfeit as determined by HPLC. The specificity was calculated as the ratio of the total number of samples that are identified as authentic by the technology to the total number of samples that are authentic as determined by HPLC.

#### Sensitivity and specificity of CD3+

CD3+ analysis (packaging print, packaging material, and dosage unit visual examination) identified all the samples that were substandard/counterfeit (sensitivity = 1.00); however, the specificity in identifying authentic medicines was only 0.53 (Table 4). Considering that the “gold standard”, HPLC analysis, was based on only the dosage unit, and not on both the dosage unit together with the package information, the study also looked at the specificity and sensitivity of the CD3+ when only the dosage unit was examined with the tool; that is, excluding the CD3+ packaging examination. The results shown in Table 5 indicate an improvement of the specificity to 0.64; the sensitivity remained unchanged at 1.00.

**Table 4:** Sensitivity and specificity data for CD3+ analysis based on packaging print, packaging material, and dosage unit

	HPLC ID and assay	
	Counterfeit/substandard	Authentic
CD3+ analysis		
Counterfeit/substandard	14	33
Authentic	0	37

	Sensitivity	Specificity
	1.00	0.53

**Table 5:** Sensitivity and specificity data for CD3+ dosage-unit-only analysis

	HPLC ID and assay	
	Counterfeit/substandard	Authentic
CD3+ dosage analysis		
Counterfeit/substandard	14	25
Authentic	0	45

	Sensitivity	Specificity
	1.00	0.64

The data for CD3+ analysis of blind samples that was performed to establish that the study participants were correctly using the CD3+ tool is shown in Table 6. Data from the four study sites (BOL, HO, KSI, and TAK) that participated in this study shows only 30 % “consistent with” (CW) results for the authentic samples. Based on the high (70 %) NCW results reported by the study sites, even for the authentic samples, CePAT performed an investigation to determine if the use of CDAIL library images contributed to the results. Using the same samples, CePAT staff compared CD3+ analyses using only library images against analyses using a side-by-side comparison of images of the authentic sample captured at the time the sample was being analysed. The CePAT data is shown in Table 6. The specificities calculated based on the CePAT data were 0.2 using library images and 1.00 using the side-by-side comparison. This limited data suggests using side-by-side comparison rather than the image library may help improve the specificity of CD3+ in determining authentic anti-malarial medicines.

**Table 6:** Comparison of CD3+ analysis of blind (authentic) samples using library images versus using side-by-side comparison of the blind sample and authentic product

Authentic sample	Study sites					
	*Based on library images*					*Side*-*by*-*side comparison* ^a^
	BOL	HO	KSI	TAK	CePAT	CePAT
Coartem-D	CW	NCW	NCW	NCW	CW	CW
Coartem	CW	NCW	NCW	CW	NCW	CW
Lumerax 40/240	NCW	NCW	NCW	NCW	NCW	CW
Winthrop 50/135	CW	NCW	NCW	CW	NCW	CW
Winthrop 100/270	NCW	NCW	NCW	CW	NCW	CW

*CW* consistent with (authentic); *NCW* Not consistent with (counterfeit/substandard)

^a^Side-by-side comparison of images of sample to that of the physical authentic sample

It is important to note that although the CD3+ identified authentic products as counterfeit the tool did not identify counterfeit products as authentic. It has also been noted by the US FDA (unpublished data) that as users become more experienced with using the CD3+ and CDAIL overtime, this increased experience has shown an improvement in the specificity of CD3+.

#### Sensitivity and specificity of the GPHF Minilab

GPHF Minilab analysis (based on TLC identification and estimation of content) identified all the authentic anti-malarial samples (specificity = 1.00), and the sensitivity in determining substandard/counterfeit products was 0.79 (Table 7). Separate treatment of the data to extract information that compared TLC identification with HPLC identification data showed that the GPHF Minilab had sensitivity (1.00) and specificity (1.00) when used only for identification (Table 8).

**Table 7:** Sensitivity and specificity data for GPHF Minilab analyses based on identity and content

	HPLC ID and assay	
	Counterfeit/substandard	Authentic
Minilab-ID and content		
Counterfeit/substandard	11	0
Authentic	3	70

	Sensitivity	Specificity
	0.79	1.00

**Table 8:** Sensitivity and specificity data for GPHF Minilab analyses based on ID Only

	HPLC ID	
	Counterfeit/substandard	Authentic
Minilab-ID only		
Counterfeit/substandard	11	0
Authentic	0	73

	Sensitivity	Specificity
	1.00	1.00

#### Sensitivity and specificity of the TruScan Raman spectrometer

The sensitivity and specificity data obtained for the TruScan Raman spectrometer compared to HPLC identification and assay data were very similar to that of the GPHF Minilab, 0.79 and 0.99, respectively (Table 9). Comparison of the TruScan Raman data to HPLC identification only data, however, showed sensitivity (1.00) and specificity (0.99) for the TruScan Raman spectrometer (Table 10).

**Table 9:** Sensitivity and specificity data for TruScan Raman spectrometer

	HPLC ID and assay	
	Counterfeit/substandard	Authentic
TruScan raman		
Counterfeit/substandard	11	1
Authentic	3	69

	Sensitivity	Specificity
	0.79	0.99

**Table 10:** Sensitivity and specificity data for TruScan Raman spectrometer—ID Only

	HPLC ID	
	Counterfeit/substandard	*Authentic*
TruScan ID only		
Counterfeit/substandard	11	1
Authentic	0	72

	Sensitivity	Specificity
	1.00	0.99

#### Counterfeit field samples^7^

Eight of the field samples collected from the study sites were confirmed by HPLC analyses to be counterfeits with zero (or almost zero) active pharmaceutical ingredient (API) content. These samples comprised seven counterfeit Coartem tablets samples and one counterfeit Ipca artemether–lumefantrine (20/120 mg) tablet sample. All three technologies—CD3+, GPHF Minilab, and TruScan Raman spectrometer—identified these eight samples as counterfeits.

#### Evaluation of the ability of the technologies to distinguish between different strengths of a product

The results of tests performed to determine the ability of the screening tools to distinguish between products presented in different strengths is provided in Table 11. The data shows the CD3+ and GPHF Minilab clearly determined the two products compared in each case were different, whereas the TruScan did not distinguish between these products as shown by the erroneous “pass” results. The results provide an indication of the capability of these tools to identify substandard products having a lower or a higher amount of active ingredient(s) than what is claimed. This suggests that the TruScan may be inadequate in detecting certain substandard medicines; hence further studies need to be done to truly assess the Raman spectrometer’s ability to detect substandard medicines.

**Table 11:** Performance of CD3+, TruScan Raman, and GPHF Minilab in distinguishing between different strengths

Brand name	Manufacturer	Sample composition/strength	Reference product	CD3+ (dosage)	TruScan raman	GPHF minilab
Winthrop	Sanofi	AS-AQ 50/135	AS-AQ, 100/270 mg, Sanofi	Fail^a^	Pass^b^	Fail
Winthrop	Sanofi	AS-AQ 100/270	AS-AQ, 50/135 mg, Sanofi	Fail	Pass	Fail
Lumerax	Ipca	AR-LU 40/240	AR-LU, 20/120 mg, Ipca	Fail	Pass	Fail
AR-LU 20/120	Ipca	AR-LU 20/120	AR-LU, 40/240 mg, Ipca	Fail	Pass	Fail

*AS-AQ* Artesunate and Amodiaquine fixed-dose combination tablets

*AR-LU* Artemether and Lumefantrine fixed dose combination tablets

^a^ *Fail* Sample is not consistent with reference product (different)

^b^ *Pass* Sample is consistent with reference product (identical)

### Objective 2 results: reliability

Reliability is the ability of a tool to produce the same results with repeated measurements. In this study, three analysts each obtained the same set of conclusions for the 11 samples used to evaluate reliability of the three tools—CD3+, GPHF Minilab, and TruScan Raman analyzer. Also, one analyst obtained consistent conclusions for all three tools when analyses of the 11 samples were repeated on three different days for each technology. The reliability of CD3+, GPHF Minilab, and TruScan Raman technologies was each determined to be 100 % in this study.

### Object 3 results: comparative advantages and disadvantages

One objective of the study was to compare the advantages and disadvantages of the three field-based screening techniques and establish complementary advantages in using more than one tool. CD3+ complements the visual inspection test which is a requirement for medicine quality evaluation. It is the only technology that provides comprehensive examination of printing style, packaging, and dosage unit and, hence, could be used in concert with other screening technologies to improve detection of counterfeit and substandard products. CD3+ results however, are user dependent, as the user decides whether sample image is consistent with that of the reference product. The user’s decision can be improved if side-by-side comparison (e.g. suspect and authentic tablets) is used for analysis rather than comparison to an image from library and as users become more experienced with using the CD3+ and CDAIL overtime. Compared to Minilab and TruScan, CD3+ has the relative advantage of being significantly less expensive, portable as a handheld tool, easy to learn and operate, and can be used to screen both dosage unit and packaging.

The TruScan Raman spectrometer was found to be very robust, as the pass/fail decisions were user independent. The tool demonstrated high sensitivity and high specificity for identification—but did not distinguish between products having as much as 50 % difference in API content. This shows that additional studies may be warranted to determine the technology’s ability to detect substandard medicines.

The GPHF Minilab uses a low-cost technology—TLC—that uses standards under the same conditions as the sample during testing. The technique has wide application and can be used for a wide range of active ingredients and does not need the development of a library of authentic product. The GPHF Minilab demonstrated high specificity in determining authentic products. Using it as an identification only tool, the GPHF Minilab demonstrated high sensitivity and specificity. TLC results, however, may be influenced by the user, especially when used for drug content or assay estimation.

Using combination of CD3+, with either the Minilab or TruScan, to screen for medicine quality will allow for complete examination of both the dosage units and the packaging information in addition to assessment of other quality attributes leading to a detailed view of the product to decide whether it is authentic or counterfeit.

## Conclusions

The study revealed the CD3+ is very effective in identifying counterfeit products as well as in identifying differences in strength of a given product. The technology is also unique in that it allows for the visual inspection of both the packaging information and the dosage unit and can detect very subtle differences in the packaging and dosage unit. Comparing authentic images side by side with samples seems to improve the detection ability of the technology than using library images. Additionally, one of the strengths of the technology is conducting real-time screening of large numbers of same items very quickly, once the product is determined to be authentic or counterfeit.

The Minilab which is based on TLC and the TruScan based on Raman spectroscopy are also powerful in determining the identity of the API in the product but do not have the ability to assess packaging information. Minilab cannot be relied upon to determine falsification of product brands. The TruScan also showed limited ability to detect differences in strength of the same product whereas the Minilab and the CD3+ were effective in detecting differences in strength of dosage unit as measured by API content.

**Fig. 1:**
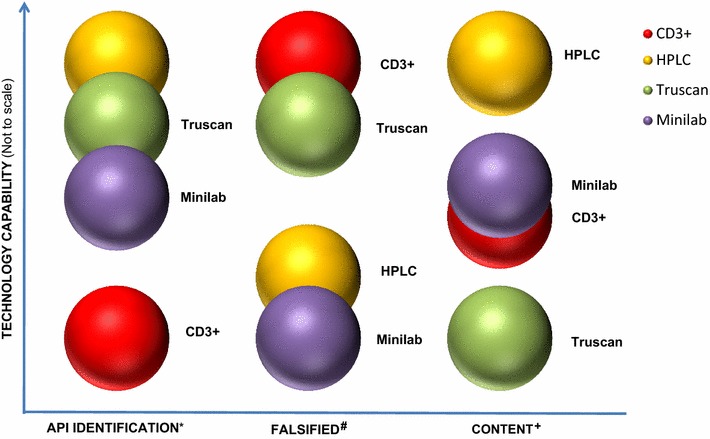
Relative effectiveness of CD3+, TruScan, GPHF minilab, and HPLC as medicine quality control tools. *Key* *Deceptive API, ^#^Deceptive product, ^+^API content

The comparative advantages of the three technologies are captured in Fig. 1—these relative advantages referred to as the effectiveness index in the Figure, is the ability of the techniques to assess various quality attributes including:

- Identity of the active pharmaceutical ingredient(s);
- Identity of the product brand, that is, whether or not a product is falsified; and,
- Determination of the API content (“strength”).

Using the tools in unison presents an excellent opportunity to assess various quality attributes of the medicine and increases the chance of detecting counterfeit and substandard medicines.

## Challenges and limitations

The CD3 Authentic Image Library (CDAIL) products were from three manufacturers (Table 1). The use of targeted manufacturers and products represents a straightforward application of the CD3 technology; however, during the development of the CDAIL for this project, it was noted that some of the authentic products presented challenges. For instance, the packaging of the authentic products from one manufacturer displayed inconsistencies/differences in the packaging information for the same product. While these variations were included in the CDAIL, it raised concerns how to account for these inconsistencies/differences to ensure that all variations in the authentic packaging information of a given product are captured and included in the CDAIL.

Another challenge encountered during the study was the number of different presentations of packaging and labelling for the same product. For example, a total of 176 anti-malarial field samples that were supposed to match the authentic samples acquired from the manufacturer were collected at the five study sites. Of these, only 73 field samples were used for the study, as visual examination of the remaining samples showed the packaging and labelling differed significantly from the authentic samples. The CD3+ library was generated using specific authentic samples; field samples that were clearly different from the authentic products were not used so as not to bias the study.

It was observed that manufacturers of the anti-malarial products presented several variations of their packaging and labelling—in some cases to provide for different languages, packaging for different age groups, packaging for multiple-dose versus unit dose, and, in one instance, even different marketing logos or manufacturer names for a company that appeared to be transitioning from one logo to another. This presents a major challenge in identifying authentic products by CD3+ because the packaging and labelling information were varied in very subtle ways that were not captured in the library. This should be a major concern particularly for regulatory authorities, as it suggests authentic products with a high degree of packaging variability present opportunities for counterfeits to enter the legitimate supply chain. If the manufacturers would standardize their packaging it would provide for a more secure supply chain. Manufacturers need to standardize packaging to ensure secure supply chain.

Also, insufficient units of the TruScan for all field sites precluded actual field sample testing using this tool, hence TruScan testing of all samples was performed at a single study site.

## Footnotes

1 The GPHF Minilab is a trademarked property of the Global Pharma Health Fund (GPHF).

2 The Thermo Scientific TruScan RM analyzer is a trademarked property of Thermo Fisher Scientific Inc.

3 Sensitivity is defined in this study as the proportion of medicines that are detected as substandard/counterfeit by the tool out of all the tablets that are substandard/counterfeit. It is the probability that the tool will correctly detect substandard/counterfeit anti-malarial tablets.

4 Specificity is the proportion of anti-malarial tablets that are identified as authentic by the tool out of all the tablets that are of authentic quality. It is the probability that the tool will correctly identify good quality anti-malarial tablets.

5 Reliability is the ability of the tool to produce the same results with repeated measurements.

6 TruScan Raman spectrometers utilize an embedded decision engine containing proprietary algorithms for the comparison.

7 Information about these counterfeit medicines were provided to the Ghana FDA.
